# Alzheimer's Disease brain‐ derived Extracellular Vesicles induce cognitive impairment in mice

**DOI:** 10.1002/alz70855_099289

**Published:** 2025-12-23

**Authors:** Lisandra Silva Pinheiro, Victor Bodart, Rudimar L. Frozza, Mayara Mendonca, Sergio T. Ferreira, Fernanda G. De Felice

**Affiliations:** ^1^ Federal University of Rio de Janeiro, Rio de Janeiro, Rio de Janeiro, Brazil; ^2^ FIOCRUZ, Rio de Janeiro, Rio de Janeiro, Brazil; ^3^ Institute of Biophysics, Federal University of Rio de Janeiro, Rio de Janeiro, Brazil; ^4^ D'Or Institute for Research and Education, Rio de Janeiro, RJ, Brazil; ^5^ Queen's University, Kingston, ON, Canada; ^6^ D’Or Institute for Research and Education, Rio de Janeiro, Brazil; ^7^ Federal University of Rio de Janeiro, Rio de Janeiro, Brazil

## Abstract

**Background:**

Alzheimer's disease (AD) is a neurodegenerative disorder and the most common form of dementia in the elderly. The deposition of β‐amyloid peptide (Aβ), a product of the processing of amyloid precursor protein (APP), and the hyperphosphorylation of Tau are the major histopathological hallmarks in the brains of AD patients. Extracellular vesicles (EVs) are lipid nanoparticles secreted by many cell types. EVs carry various biomolecules from their cell of origin and can cross tissue barriers. EVs have been implicated in the spread of neuropathology in AD, but their role in the behavioral outcomes associated with AD remains unclear.

**Method:**

EVs isolated from post‐mortem brain tissue of control, AD, and 12‐month‐old APP/PS1 mice were injected into the hippocampi of 7‐month‐old wild‐type (WT) mice. Memory was assessed using behavioral tests.

**Results:**

Our results suggest that C57BL/6 mice injected with brain‐derived EVs did not show motor or behavioral deficits. However, EVs derived from AD brains induced cognitive impairment in mice after 14 and 21 days of injection, indicating that AD‐EVs and APP/PS1‐EVs trigger memory deficits in WT mice.

**Conclusion:**

These findings demonstrate that EVs can carry pathological molecules associated with AD and induce memory impairment that mimics features of the disease. This evidence positions EVs as an important target for AD research and highlights their potential as a novel target for therapeutic strategies in neurodegenerative diseases.

